# Antitumor Immune Response Triggered by Metal-Based Photosensitizers for Photodynamic Therapy: Where Are We?

**DOI:** 10.3390/pharmaceutics13111788

**Published:** 2021-10-26

**Authors:** Alain C. Jung, Fabien Moinard-Butot, Chloé Thibaudeau, Gilles Gasser, Christian Gaiddon

**Affiliations:** 1 Laboratory “Streinth”, UMR_S 1113 IRFAC, Université de Strasbourg-Inserm, 67200 Strasbourg, France; fabmb8764@gmail.com (F.M.-B.); chloe.thibaudeau85@gmail.com (C.T.); 2 Laboratoire de Biologie Tumorale, Institut de Cancérologie Strasbourg Europe, 67200 Strasbourg, France; 3 Département d’Oncologie Médicale, Institut de Cancérologie Strasbourg Europe, 67200 Strasbourg, France; 4 Laboratory for Inorganic Chemical Biology, Institute of Chemistry for Life and Health Sciences, CNRS, Chimie ParisTech, PSL University, 75005 Paris, France; gilles.gasser@chimieparistech.psl.eu

**Keywords:** cancer, photodynamic therapy, transition metals, immunogenic cell death

## Abstract

Metal complexes based on transition metals have rich photochemical and photophysical properties that are derived from a variety of excited state electronic configurations triggered by visible and near-infrared light. These properties can be exploited to produce powerful energy and electron transfer processes that can lead to oxygen-(in)dependent photobiological activity. These principles are the basis of photodynamic therapy (PDT), which is a clinically approved treatment that offers a promising, effective, and noninvasive complementary treatment or even an alternative to treat several types of cancers. PDT is based on a reaction involving a photosensitizer (PS), light, and oxygen, which ultimately generates cytotoxic reactive oxygen species (ROS). However, skin photosensitivity, due to the accumulation of PSs in skin cells, has hampered, among other elements, its clinical development and application. Therefore, these is an increasing interest in the use of (metal-based) PSs that are more specific to tumor cells. This may increase efficacy and corollary decrease side-effects. To this end, metal-containing nanoparticles with photosensitizing properties have recently been developed. In addition, several studies have reported that the use of immunogenic/immunomodulatory metal-based nanoparticles increases the antitumor efficacy of immune-checkpoint inhibitor-based immunotherapy mediated by anti-PD-(L)1 or CTLA-4 antibodies. In this review, we discuss the main metal complexes used as PDT PSs. Lastly, we review the preclinical studies associated with metal-based PDT PSs and immunotherapies. This therapeutic association could stimulate PDT.

## 1. Introduction

The use of metal complexes as pharmaceutical drugs is widespread in medicine, especially for the management of patients with cancer. Cisplatin and its derivatives are employed in almost 50% of chemotherapeutic treatments against cancer to induce cytotoxic activity by generating DNA damages. Despite this broad use, platinum salts have limitations caused by the presence of inherent or induced resistance mechanisms, such as mutations in p53 [1]. However, metal-based compounds have a biological and chemical diversity distinct from that of organic drugs, which drives their attractiveness in the search for new therapeutics with new mechanisms of action for treating cancers [2]. They offer a wide range of oxidation states and variable geometries. The structural and electronic properties of transition metal complexes can be tailored by altering the identity of the metal and its oxidation state. The aim is to induce changes in physical properties and chemical reactivities such as charge, solubility, ligand exchange rates, metal–ligand binding forces, redox potentials at the base metal and ligand, and ligand conformations [3,4,5,6]. In addition, ligands can be modified to contribute to biological activity [7,8]. In this context, the use of transition metal (e.g., ruthenium and osmium) complexes is increasing due to the intrinsic characteristics of the metal atom that has a partially filled *d* subshell or which can give rise to cations with an incomplete *d* subshell [9,10,11,12]. This characteristic may provide interesting photophysical and chemical properties, which include strong luminescence, high chemical and photophysical stability, and high production of singlet oxygen upon light irradiation, which is particularly relevant for photodynamic therapy (PDT) [13,14,15,16,17].

In this review, we summarize key information on the major metal-based PSs depending on their known modes of action (genotoxic vs. DNA-independent cytotoxicity), present the efforts made to improve a more targeted delivery of PSs, and discuss in more detail their interest in modulating the immune antitumor response via their pro-immunogenic properties. Importantly, this review does not aim to present an exhaustive catalog of metal-based PSs, but specific examples that were chosen to illustrate the points discussed below.

## 2. Photodynamic Therapy

**Figure 1:**
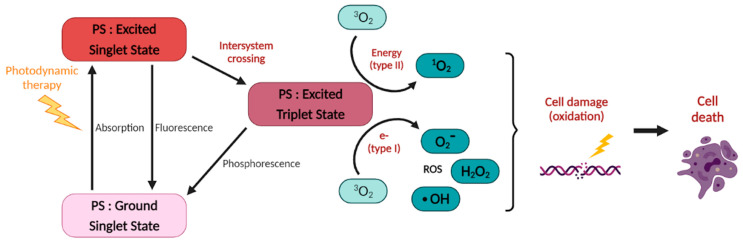
Mechanisms of action of photodynamic therapy (PDT).

By definition, PDT uses a light-activable chemical, the “PS”, whose cytotoxic activity requires both activation by light, usually in the visible spectrum, and the presence of oxygen to produce singlet oxygen (^1^O_2_) and/or other reactive oxygen species (ROS) [18]. More specifically, the PDT effect relies on the excitation of the PS to first reach a singlet excited state that then undergoes intersystem crossing (ISC) to reach a triplet state (Figure 1). This latter sensitizes cytotoxic singlet oxygen (^1^O_2_) through a Type II energy transfer or participates in Type I electron transfer reactions to generate other reactive oxygen species (ROS, e.g., hydroxyl radicals and superoxide radicals). The Type II mechanism is accepted as the predominant pathway for most of the currently approved PSs. For a more detailed review about the basic principles of PDT, see [19] and the references therein.

**Figure 2:**
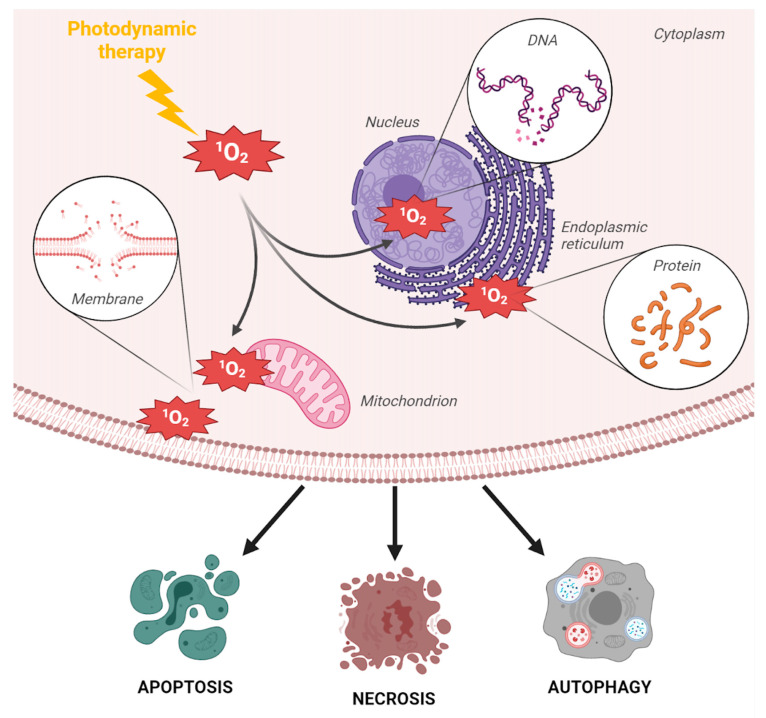
Cellular damages induced by PDT and triggered cell death types. The cytotoxicity of PDT relies on the generation of ^1^O_2_ singlet oxygen and on the subsequent oxidation of different cell constituents leading to (i) DNA base oxidation in the nucleus, resulting in DNA cleavage, (ii) accumulation of misfolded proteins in the endoplasmic reticulum, triggering endoplasmic reticulum stress and the unfolded protein response, (iii) mitochondria oxidative stress, resulting in perturbation of the mitochondrial membrane potential and the release of proapoptotic proteins in the cytoplasm, or (iv) oxidation of phospholipids, which perturbs the permeability and/or the integrity of the plasma membrane (increased influx of ions and increased efflux of cell content). Depending on several parameters (chemical nature of the PS, preferential intracellular localization of the PS after cellular uptake, and cell/tissue context), PDT can cause cell death via different routes, including necrosis, apoptosis, and autophagy.

The singlet oxygen-driven cytotoxicity during PDT mainly relies on oxidation mechanisms that lead to the degradation of amino acids, certain DNA nucleic bases, and lipids composing cell membranes and the mitochondria, which ultimately triggers different kinds of cell death, including necrosis, apoptosis, paraptosis, and autophagy [20,21,22] (Figure 2).

The light-dependent cytotoxicity of the PS is, therefore, at the base of the principle of PDT, which usually relies on the systemic intravenous injection of the PS. However, activation of the PS is normally made locally by illumination with low-powered laser light (the exception being daylight PDT [23]). The advantages of PDT over other therapies are, therefore, the high spatiotemporal control and the low systemic toxicity of the treatment. PDT currently has several indications in different cancer types, including obstructing esophageal cancer, locally advanced, non-curable head and neck squamous cell carcinoma (HNSCC), non-small-cell lung cancer, prostate cancer, and superficial basal cell carcinoma [24,25,26,27,28] (Table 1). The main PSs used clinically are derivatives of porphyrin. However, organic PSs may display poor water solubility and/or their maximum excitation wavelength may not penetrate deeply enough in tissues, making them unsuitable for the treatment of deep-seated cancer lesions [29]. Metal-based PSs, therefore, offer an interesting flexibility of ligands that can potentially improve these issues. Moreover, a current limitation of PDT is the skin photosensitivity caused by the accumulation of a PS in skin cells [30]. The use of nanoparticles that allows for a more specific delivery of PSs to tumor cells has been developed in recent years to address this limitation [31,32].

## 3. Major Metal-Based Photosensitizers and Their Mode of Action

The most studied transition metal complexes used as PDT PSs [33] are currently based on ruthenium(II) polypyridyl complexes [34,35], platinum(IV), and rhodium(III) [36,37,38] followed, more recently, by iridium(III) [39,40], rhenium(I) [41], and osmium(II) [42] or even a combination of transition metals [43]. Several reviews discussed this topic [44,45].

As mentioned above, the generation of ^1^O_2_ upon light excitation is responsible for the oxidation of cell constituents and subsequent cell death. However, the precise molecular mechanisms that underlie metal-based PDT cytotoxicity may change depending on the metal; some transition metals have been reported to interact with DNA and provoke DNA damage upon PDT, whereas others trigger cell death via mitochondria or endoplasmic reticulum stress (Figure 2).

**Figure 3:**
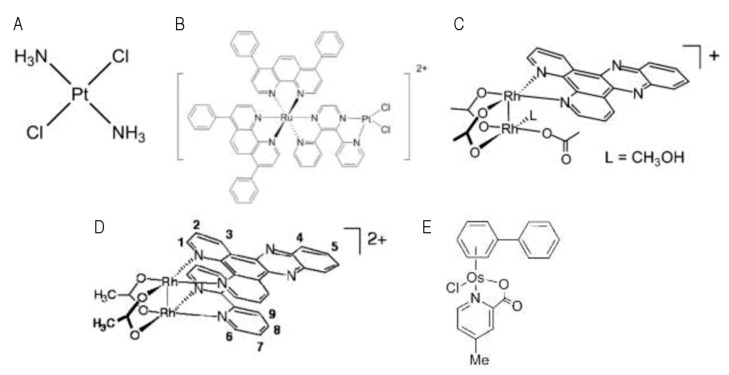
Examples of transition metal-based compounds that display genotoxic activity upon PDT. (**A**) Structure of transplatin. (**B**) Structure of the [(Ph_2_phen)_2_Ru(dpp)PtCl_2_]^2+^ ruthenium(II)–Pt(II) dinuclear complex (Used with permission of ROYAL SOCIETY OF CHEMISTRY, from [64]; permission conveyed through Copyright Clearance Center). (**C**) Structure of the *cis*-[Rh_2_(μ-O_2_CCH_3_)_2_(dppz)(η1-O_2_CCH_3_)(CH_3_OH)](O_2_-CCH_3_) dirhodium complex studied in [53] (Adapted with permission from [53]. Copyright © 2021 American Chemical Society). (**D**) Structure of the *cis*-[Rh_2_(μ-O_2_CCH_3_)_2_(bpy)(dppz)]^2+^ dirhodium complex studied in [58] (Adapted with permission from [58]. Copyright © 2021 American Chemical Society). (**E**) Structure of the [(η6-biphenyl)Os(4-methyl-picolinate)Cl] Os-based complex studied in [62] (Adapted with permission from [62]. Copyright © 2021 American Chemical Society).

Examples of transition metals that display a genotoxic activity upon light include Platinum (Pt), Rhodium (Rh), and Osmium (Os). Luminescent Pt complexes have been explored due to their photophysical properties [46,47,48]. Transplatin (Figure 3A), a nontoxic isomer of cisplatin, was reported to show increased toxicity upon UVA irradiation of HaCaT keratinocytes, as well as A2780 and A2780CIS ovarian carcinoma cells. This has been attributed to the loss of chloride ligands and the formation of bifunctional DNA inter-strands and DNA–protein crosslinks that are unable to form in the dark [49]. Brewer et al. reported a ruthenium(II)–Pt(II) dinuclear complex (Figure 3B), where the dichloro-platinum moiety is attached to a dpp ligand (2,3-bis(2-pyridyl)pyrazine). Irradiation caused hydrolysis of chlorides and binding to plasmid DNA in vitro [50]. However, the results presented in these studies require confirmation in a cellular context. Historically, Rh_2_ complexes have been shown to display a natural ability to bind to a DNA duplex and to inhibit DNA replication [51,52]. Interestingly, Angeles-Boza et al. reported the synthesis of dirhodium complexes, one of which (Figure 3C) displayed increased in vitro DNA binding and cleavage abilities upon irradiation with visible light [53]. These examples show that light activation of metal complexes may lead to cytotoxicity via the liberation of a specific part of the metal complex that will exert biological activity, such as DNA binding, which is independent of the production of ROS. These metal complexes are defined as photo-activated chemotherapy (PACT) agents [54,55,56,57].

However, the frontier between chemicals used for PDT and chemicals used for PACT is relatively plastic. For instance, Angeles-Boza’s group also synthesized an Rh_2_ heteroleptic complex (*cis*-[Rh_2_(μ-O_2_CCH_3_)_2_(bpy)(dppz)]^2+^; Figure 3D), which was found to display both O_2_-dependent and O_2_-independent cytotoxicity, in proportions that are comparable to the classical hematoporphyrin PDT PS [58]. Oxygen-independent DNA photocleavage by dirhodium complexes upon excitation with visible light has also been reported [59]. In this case, unlike traditional PDT that relies on the interaction between the activated PS and oxygen, irradiation of *cis*-[Rh_2_(μ-O_2_CCH_3_)_2_(CH_3_CN)_6_]^2+^ with visible light promotes the exchange of two CH_3_CN ligands with H_2_O water molecules. The resulting species covalently bind to DNA and are more cytotoxic than the starting material, resulting in a 34-fold increase in the IC_50_ value against human skin Hs-27 cells exposed to visible light compared to those incubated in the dark [60]. Os-derived complexes display in vitro antitumor activity in several cancer cell lines models (including 1205 Lu melanoma cells and A2780 ovarian cells), as well as interesting reactivity toward DNA [61,62,63] (e.g., [(η6-biphenyl)Os(4-methyl-picolinate)Cl] is shown in Figure 3E). These compounds were shown to accumulate in the mitochondria, nucleolus, and the nuclear membrane of A2780 ovarian cancer cells, form DNA adducts, and seemingly trigger morphological changes (plasmic membrane blebbing and nucleus condensation) that are compatible with apoptosis.

Even if these metal-based compounds may represent potential alternatives to platinum-based compounds already used in the clinic, considering the condition that they induce fewer side-effects, they still have all the inherent limitation of targeting DNA. Indeed, as with cisplatin or oxaliplatin, by targeting DNA, their efficacy is likely to be limited by mutations or alterations in pathways involved in detecting and repairing DNA damage or eliminating damaged cells. For instance, the induction of apoptosis upon DNA damage or other cell stress is frequently compromised in most human tumors, due to mutations that affect the *TP53* tumor suppressor gene and drive resistance to genotoxic therapies [65,66]. Interestingly, metal-based PDT has been reported to be able to trigger *TP53*-independent cell death, through the induction of the endoplasmic reticulum (ER) stress. ER stress is commonly triggered by the accumulation of misfolded proteins in the ER lumen, which induces the activity of signaling pathways depending on ER membrane resident proteins (inositol-requiring enzyme 1-a (IRE1-a), activating transcription factor 6 (ATF6), and PKR-like ER kinase (PERK)). The subsequent activation of the unfolded protein response (UPR) either allows the restauration of protein homeostasis or triggers apoptotic cell death in case of prolonged ER stress [67].

**Figure 4:**
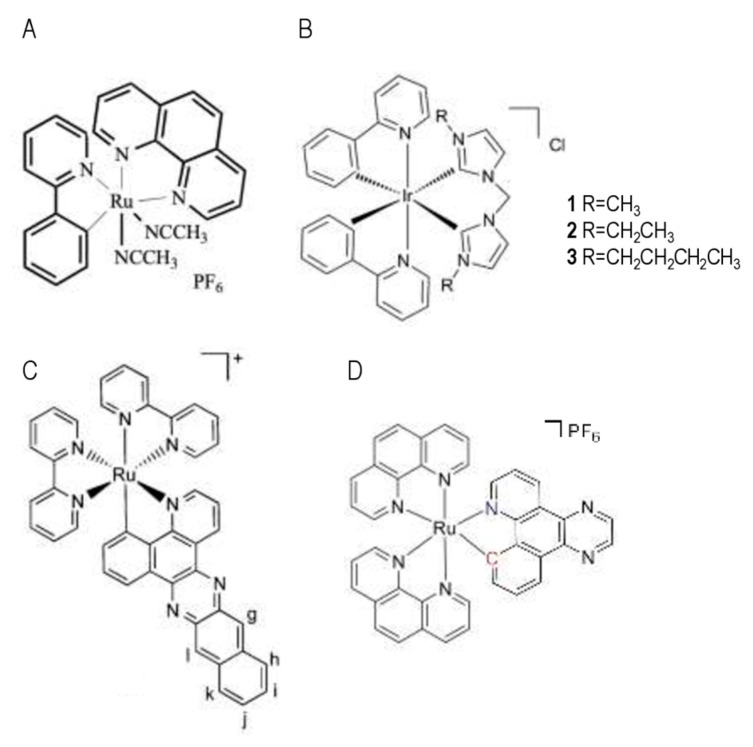
Examples of transition metal-based compounds that can trigger nongenotoxic cytotoxicity upon PDT. (**A**) Structure of ruthenium-derived Compound 11, studied in [69,70]. (**B**) Structures of three cyclometalated iridium(III) complexes studied in [72] (Adapted with permission from [72]. Copyright © 2021 Elsevier Ltd. All rights reserved). (**C**) Structure of the [Ru(bpy)_2_(pbpn)]PF_6_ ruthenium-based complex studied in [73] (Adapted with permission from [73]. Copyright © 2021 American Chemical Society). (**D**) Structure of the [Ru(bpq)(phen)_2_]PF_6_ ruthenium-based complex studied in [17].

Examples of transition metal complexes that can trigger non-genotoxic cell death include ruthenium (Ru), iridium (Ir), and osmium (Os) complexes. For instance, Meng et al. were the first to show that Ru-based organometallic complexes (e.g., ruthenium-derived Compound 11 is shown in Figure 4A) exert, without activation by light, their cytotoxicity via the induction of the ER stress pathway in glioblastoma and colon cancer cells, despite showing some ability to interact with DNA [2,8,68,69,70]. Activation of the ER stress effector CHOP was shown to be necessary to induce cytotoxicity. Similarly, the lipophilicity of cyclometalated Ir(III) complexes was found to correlate with cellular uptake and cytotoxicity in the dark, and they were found to preferentially accumulate in the ER of HeLa cells and cause ER stress (induction of the expression of the *CHOP* pro-apoptotic gene), which resulted in a disturbed mitochondrial morphology and function, ultimately initiating an intrinsic apoptotic pathway [71]. Li and collaborators synthesized cyclometalated Ir(III) complexes carrying *N*-heterocyclic carbene ligands (see Figure 4B for examples), and they showed a correlation between lipophilicity and uptake by cervical cancer HeLa cells. Moreover, their data show that these complexes accumulate into mitochondria. This study uncovered mechanisms that induce mitochondrial damages, ROS production, cytochrome c release, caspase-3 and PARP cleavage, and apoptotic cell death, with no disruption of the cell cycle and no genotoxicity [72].

Hence, one of the interesting questions is whether the mode of action of metal-based compounds is identical or shows at least some similarities, when applied in the dark and when illuminated. In this regard, McFarland et al. studied the luminescent properties and cytotoxic activity of four Ru(II) complexes. Three of them were highly cytotoxic in the dark (IC_50_ values = 1–2 mM) on the HL-60 promyelocytic leukemia and SK-MEL-28 malignant melanoma cell lines, whereas one compound (with the larger π system) showed increased activity upon irradiation with visible light (IC_50_ < 1 mM) and no toxicity in the dark (IC_50_ > 300 mM) [73]. This latter compound (Figure 4C) was found to generate superoxide O_2_^−^ upon illumination and to provoke DNA aggregation/precipitation in vitro, although its ability to provoke DNA damages seemed limited [73]. In the AGS and KATO III gastric cancer cell lines, Solis-Ruiz et al. showed that Ru(II) polypyridyl complexes bearing increased π-conjugation on the cyclometalated ligand were highly cytotoxic upon light irradiation (IC_50_ < 1 mM) [17]. The authors showed that the mode of action of Ru(II) polypyridyl-based PSs (e.g., [Ru(bpq)(phen)_2_]PF_6_ is shown in Figure 4D) depends on the compound structure; for example, generation of DNA double strand breaks and activation of caspase-3-dependent apoptosis were observed with compounds bearing an Ru–C bond, but not in compounds bearing only Ru–N bonds. Lastly, of high interest, Ru(II) polypyridyl complex-based PSs triggered cell death independently of the p53 status of the cell lines (AGS cells have a wild-type *TP53* gene, whereas KATO III cells harbor a *TP53* deletion). PDT using Ru(II) polypyridyl as PSs might, therefore, be particularly relevant for the treatment of tumors with a mutated and/or deleted *TP53* gene, which are known to be responsible for resistance to most genotoxic anticancer therapies.

**Figure 5:**
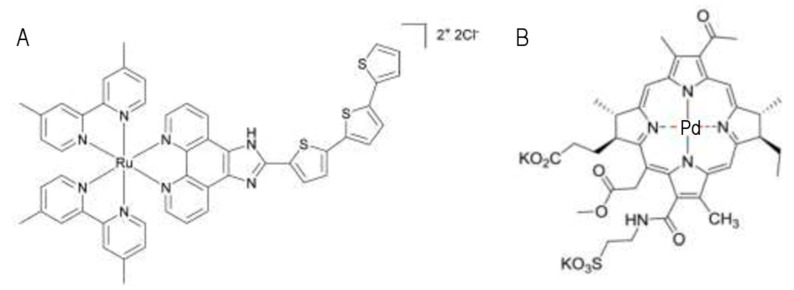
Examples of transition metal-based compounds that are clinically approved for PDT. (**A**) Structure of TLD-14-33. (**B**) Structure of TOOKAD^®^ soluble (Padeliporfin, WST11).

Hence, these properties of metal-based compounds that can exert their cytotoxicity outside the requirement of DNA interaction and activation of p53 represent a competitive advantage for their clinical use. McFarland and Gasser recently discussed the metal-based PSs that made it to clinical trials [33]. Despite the large variety of Ru(II) polypyridyl complexes that have been investigated (not covered in this review), TLD-1433 [Ru(II) (4,40-dimethyl-2,20-bipyridine[dmb]) 2(2-[20,200:500,200-terthiophene]-imidazo[4,5-f][1,10]phenanthroline)]^2+^) is the only Ru-based PDT PS that has advanced to clinical trials to date. TLD-1433 (Figure 5A) has high water solubility, very low photobleaching, and selectivity toward malignant cells, including bladder cancer and leukemia cell line models [19,74,75]. In vitro and in vivo studies on TLD-1433-mediated PDT have demonstrated high therapeutic efficacy against models of bladder cancer [76]. Interestingly, TLD1433 clearance was shorter than the clearance of Photofrin (traditional, non-metal-based PS used in PDT), with comparable toxicity and pharmacokinetics, with the notable exception of skin photosensitivity. The authors proposed that TLD1433-based PDT selectivity for cancer cells relies on its higher accumulation in cancer cells due to a higher expression of the transferrin receptor compared to surrounding healthy tissue. TLD1433 has completed human phase I (ClinicalTrials.gov Identifier: NCT03053635; completed) and phase II (ClinicalTrials.gov Identifier: NCT039451625; currently recruiting) clinical trials for the management of high-risk non-muscle-invasive bladder cancer. In addition to the TLD1433 Ru complex, another metal-based PS is used in clinic. TOOKAD^®^ Soluble (Padeliporfin, WST11; Figure 5B) is the first and only palladium-based PS to be approved and is currently being used to treat low-risk prostate cancer with vascular targeted PDT. It is a negatively charged derivative of the photosynthetic pigment bacteriochlorophyll a (Bchl), a molecule that certain bacteria use to produce energy from sunlight [77]. Metal incorporation into the macrocycle changes Bchl’s hydrophobicity, optical spectrum, redox potentials, and overall reactivity compared to free Bchl [78]. Importantly, metalation also serves to stabilize the PS with no significant effects on its absorption profile [78] and increases the photodynamic activity. Padeliporfin vascular-targeted photodynamic therapy is a safe, effective treatment for low-risk, localized prostate cancer [27].

## 4. Immunogenicity of Targeted Metal-Based PDT: Therapeutical Associations with Immunotherapies

**Figure 6:**
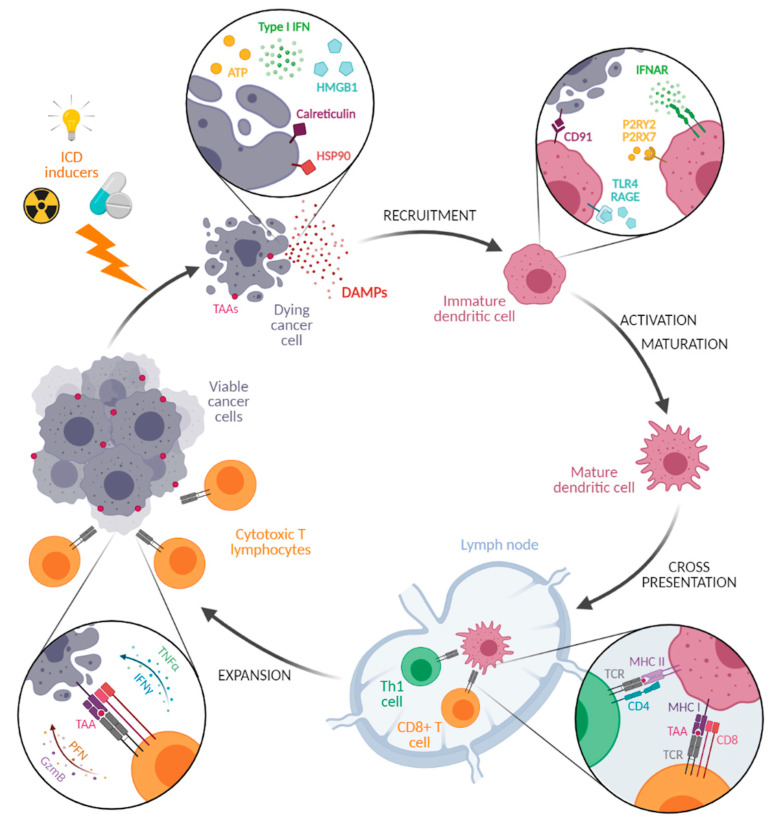
Immunogenic cell death. Treatment of tumors with immunogenic cell death (ICD) inducers, which include PDT, triggers the release of danger-associated molecular patterns (DAMPs) by dying cancer cells. DAMPs include the plasma membrane exposure of the endoplasmic reticulum calreticulin and heat-shock protein 90 (HSP90) chaperones, the extracellular release of adenosine triphosphate (ATP) and high-mobility group box 1 (HMGB1), and the secretion of type I interferon (IFN). Through their interaction with their respective receptors (including cluster of differentiation 91 (CD91), purinergic receptor P2Y2 (P2RY2)/purinergic receptor P2X7 (P2RX7), Toll-like receptor 4 (TLR4), receptor for advanced glycation end product (RAGE), and interferon-α/β receptor (IFNAR), DAMPs promote the recruitment, the maturation, and the activation of antigen-presenting cells such as dendritic cells, which engulf cell debris with tumor-associated antigens (TAAs). After migration in lymph nodes, activated dendritic cells cross-present internalized TAA on major histocompatibility complex (MHC) molecules. TAAs loaded on MHC I are presented to the T-cell receptor (TCR) expressed on CD8-positive T lymphocytes (CD8^+^ T cells), whereas antigenic peptides loaded on MCH II are presented to the TCR of CD4-positive T helper 1 lymphocytes (Th1 cells), leading to the induction of an adaptive immune response characterized by the activation of CD8^+^ T-cell lymphocyte proliferation and of their cytotoxic functions. This ultimately results in the migration of CD8^+^ T cells to the tumor site, where they provoke the death of TAA-presenting cancer cells via the secretion of antitumor cytokines (tumor necrosis factor alpha (TNFα) and INFγ), as well as perforin (PFN) and granzyme B (GzmB).

The anticancer activity of PDT relies both on direct cancer cell cytotoxicity [18] and on generating vascular damage (ischemia) [20] and creating a local inflammatory reaction [21,22]. Indeed, the oxidative stress caused by PDT induces the expression of several proinflammatory cytokines (including tumor necrosis factor-α (TNF-α), interleukin-1 (IL-1), and interleukin-6 (IL-6)), as well as the activation of innate immune cells such as macrophages, monocytes, and dendritic cells (DCs) [79]. In addition, PDT has been reported to trigger an immunogenic cell death (ICD; Figure 6), whose initial steps involve the emission of danger-associated molecular patterns (DAMPs) by dying cells, including the plasmic membrane relocalization of the calreticulin (CALR) and heat-shock protein 90 (HSP90) chaperones, the secretion of high-mobility group box 1 (HMGB1) and adenosine triphosphate (ATP), or the production of type I interferon (IFN). DAMPs further promote the recruitment, the maturation, and the activation of antigen-presenting cells (APCs) such as DCs, which mediate the presentation of tumor antigens to effector CD8 T lymphocytes, the selection and activation of antigen-specific T lymphocytes, and the activation of an adaptative memory immune response [80,81,82]. ICD is, therefore, a modality of cell death that stimulates innate and adaptive immune responses leading to generation of long-term immunological memory. Importantly, this capacity of PDT to impact tumor cell immunogenicity appears particularly relevant in the era of immunotherapies. The monitoring of these immune cells withing the tumor is a challenge that can be also addressed by imagery, including via the use of metal-based compounds [83].

**Figure 7:**
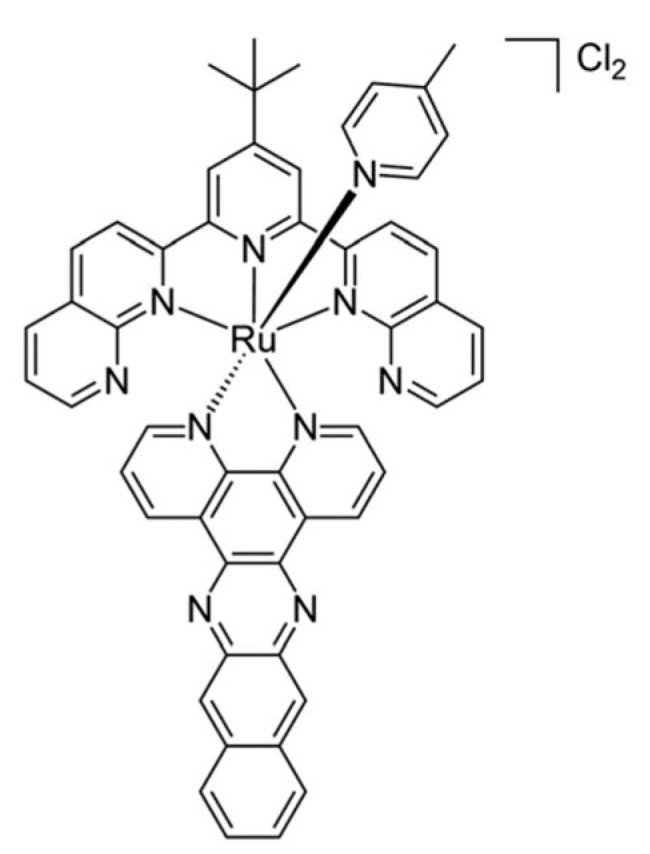
Structure of Ru(tpbn)(dppn)(4-pic)]Cl_2_. McFarland and collaborators [85] reported the synthesis of a collection of several near-infrared (NIR)-absorbing Ru(II)-based PSs. Among these compounds, Ru(tpbn)(dppn)(4-pic)]Cl_2_ was found to display one of the largest ^1^O_2_ yields and to be photocytotoxic in vitro on A375 and B16F10 melanoma cells, with a high photocytotoxic index. More interestingly, Ru(tpbn)(dppn)(4-pic)]Cl_2_-based PDT was found to have robust antitumor activity in vivo on B16F10 mice melanoma cell line models via the induction of an immunogenic apoptotic cell death, accompanied by the expression of proinflammatory cytokines, as well as of factors involved in the type I IFN pathway or in antigen presentation, and the extracellular release of ATP and HMGB1. Used with permission of ROYAL SOCIETY OF CHEMISTRY, from [85]; permission conveyed through Copyright Clearance Center.

The participation of the immune system in the response to the photodynamic effects was initially shown in 2012 by the team led by Agostinis, who used a reference model of immunocompetent mice vaccinated with cancer cells treated with hypericin (organic PS)-based PDT, to demonstrate for the first time the immunogenic nature of PDT-induced tumor cell death [84]. The immunogenic nature of PDT was also demonstrated with metal-based PSs. For instance, McFarland and coworkers reported the design and synthesis of what they propose to be an optimal combination of ligands and achieved new near-infrared (NIR)-absorbing Ru(II)-based PSs [85]. PDT using one of these Ru complexes ([Ru(tpbn)(dppn)(4-pic)]Cl_2_; Figure 7) displayed potent in vivo antitumor activity on the B16F10 mice melanoma cell line model, and it was found to elicit the expression of genes involved in the type I IFN pathway or in antigen presentation, as well as proinflammatory cytokines, and the emission of DAMPs in vitro [85,86]. In addition, vaccination experiments carried out on syngeneic mouse models (i.e., murine cancer cells grafted to immunocompetent animals) showed that one of these compounds activated by PDT provoked ICD and prophylactic protection against tumor growth. This latter feature is of particular interest in the era of immunotherapies.

If metal-based compounds show interesting properties by inducing ICD and DAMP emission by cancer cells, they may also have the same effect in noncancerous cells. In addition, the efficiency of PDT is limited in tumors with poor oxygenation. Hence, important efforts have been made in order to bypass these limitations by increasing PS selectivity for cancer cells, and by reducing the PDT requirement for oxygen within the tumor.

For instance, several strategies have been used to improve the targeted delivery of PSs to cancer cells and avoid skin photosensitivity, as well as stimulate the immunogenic nature of metal-based nanoparticle PDT. Nanoparticulate systems can enhance delivery of small-molecule drugs and biologics to tumor cells via the enhanced permeability and retention (EPR) effect by taking advantage of leaky blood vessels and reduced lymphatic drainage in tumors [87,88,89]. However, the EPR effect is under intense debate, and observations in in vivo models fail to find their equivalent in patients [90]. Thus, in order to target cancer cells more specifically, Cai et al. used a PDT compound containing hyaluronic acid (HA). HA is a ligand of the CD44 receptor, whose expression level was reported to be significantly higher in several cancers compared to healthy cells, which makes it an interesting potential carrier to more specifically deliver drugs to cancer cells [91]. The authors designed and synthesized nMOFs through the auto-assembly of the *meso*-tetra(4-carboxyphenyl)porphine (H_2_TCCP) photosensitizer and zirconium ions. This porous nanoparticle was further coated with HA, as well as with unmethylated cytosine–phosphate–guanine (CpG), which are Toll-like receptor-9 (TLR-9) agonists, in order to stimulate the maturation of DCs. The addition of CpG to the nanocarrier did not interfere with ROS generation upon irradiation with a 670 nm laser. These PCN–ACF–CpG@HA nanoparticules were taken up by H22 mouse hepatocellular carcinoma cells in a CD44-dependent manner, and a dose-dependent cytotoxicity of PCN–ACF–CpG@HA-based PDT in vitro was observed. Using a coculture approach with transwell chambers, Cai and collaborators showed that H22 cells treated with PCN–ACF–CpG@HA and PDT simulated the maturation of DCs. Furthermore, they showed that PCN–ACF–CpG@HA-based PDT treatment in vivo of H22 tumor-bearing mice resulted in a drastic tumor shrinkage associated with an increase in the number of mature DCs in tumor draining lymph nodes, increased expression of the IFN-γ, TNF-α, and IL-12p70 immune-related proinflammatory cytokines (secreted by DCs), and increased infiltration of tumor tissue by CD8^+^ and CD4^+^ lymphocytes [92].

Using a similar approach, Ni et al. designed and synthesized a cationic nanoscale metal–organic framework (nMOF) based on dinuclear W^VI^ unit and 5,10,15,20-tetra(*p*-benzoato)porphyrin (TBP) ligands used as PSs [93]. These nanocarriers were further loaded with CpG. W-TPB nMOFs were found to have a potent antitumor activity in vivo on lymphoblastoid TUBO cells, where PDT induced a significant tumor regression, accompanied by the induction of tumor infiltration by macrophages and DCs, the maturation of DCs, and an increase in systemic levels of inflammatory cytokines (IFN-α and IL-6). Interestingly, using bilateral TUBO tumor models (i.e., tumors were grafted subcutaneously on both flanks of BALB/c immunocompetent mice), the authors showed that the PDT-based treatment of one tumor also induced the regression of the distant tumor (abscopal effect), suggesting that CpG loading on the W-TBP nMOF stimulated antigen presentation by DCs and the mobilization of a memory adaptative immune response at a systemic level, and they proposed that W-TBP nMOF-based PDT has an antimetastatic effect.

Since PDT-induced ICD allows for the recruitment of cells from the immune system to facilitate the removal of cancer cells, the association of PDT with different immunotherapy modalities approved for the management of patients has been tested in animal models. For example, immune-checkpoint inhibitors (ICIs) that target programmed death ligand-1 (PD-L1) or cytotoxic T lymphocyte-4 (CTLA-4) restore the cytotoxic activity of lymphocytes against the tumor, and preclinical studies recently demonstrated that ICIs synergize with the therapeutic effect of PDT. He et al. reported the design of auto-assembling nanoscale coordination polymer (NCP) nanoparticles loaded with oxaliplatin and coated with the photosensitizer pyrolipid [94]. The authors showed that PDT triggers the exposure of CALR at the plasma membrane of CT26 mouse colon cancer cells in vitro and has an effective prophylactic tumor vaccination effect in syngeneic mouse models. Most importantly, using CT26 and MC28 bilateral tumor models, they showed that PD-L1 blockade had a synergistic abscopal effect with NCP@pyrolipid-based PDT, meaning that the combination therapy induced the regression of the primary tumors (right, irradiated tumor) and the distant tumors (left, unirradiated tumor), together with an increased infiltration of both primary and distant tumors with CD8^+^ T cells, while it increased infiltration of distant tumors only with CD45^+^ leukocytes and CD4^+^ T cells. Zhang et al. used a photosensitizer benzoporphyrin-based nanoparticle metal–organic framework (TBP-nMOF) bound to zirconium. TBP-nMOF showed stronger infrared luminescence than traditional porphyrin-based MOFs, and it generated much higher amounts of singlet oxygen even with low oxygen concentrations. The benzoporphyrin-containing nMOF induced apoptosis of murine 4T1 breast cancer cells, as well as stimulated a sharp increase in the number of CD8^+^ and CD4^+^ T cells infiltrating tumors. Its combination with an anti-PD1 antibody led to complete tumor elimination without recurrence in mice carrying 4T1. The combination allowed lymphocyte infiltration and inhibition of 4T1 tumor metastasis [95].

As indicated above, efforts have also been made to overcome tumor hypoxia, which is a resistance mechanism to both PDT (due to the low tissue O_2_ concentration) and immunotherapies (for a review, see [96] and the references therein); researchers have developed oxygen carriers including iron(III) oxide clusters or manganese dioxide (MnO_2_). Lin et al. integrated the benzoporphyrin PS with Fe_3_O as metal clusters in the core of porous nMOFs. Upon light excitation in hypoxic conditions, Fe_3_O catalyzes the formation of O_2_ from intracellular H_2_O_2_ via a Fenton-like reaction, which is further converted to singlet oxygen by excited benzoporphyrin. PDT using this nMOF proved to be effective both in vitro and in vivo on CT26 cells. In addition, this nanoparticle also triggered cell surface exposure of CALR, as well as an abscopal effect in tumor-bearing mice, which likely increased the efficacy of ICIs through a more important recruitment of CD4^+^ and CD8^+^ cytotoxic T-cell populations in the tumors [97]. In a recent study, an alternative strategy relied on the generation of MnO_2_@Chitosan-CyI (MCC) nanosystems, by adsorbing chitosan and an iodinated derivative of cyanine dye (ICy) on MnO_2_ nanoparticles [96]. ICy is derived from the FDA-approved indocyanine green and has a singlet oxygen quantum yield of 75% under NIR activation. Improved ROS production and oxygen release was observed upon NIR PDT. The authors noted an acute mobilization of the immune response in vivo, with a remarkable shrinkage of tumors upon PDT, a higher infiltration of tumors with CD4^+^ and CD8^+^ lymphocytes, a higher infiltration of tumor-associated macrophages (TAMs), and a polarization of these TAMs toward the antitumoral M1 subtype, as well as a strong abscopal effect. The hypothesis for the mode of action of MCC-based PDT is that MnO_2_ decreases the cellular levels of glutathione and serves as an oxygen source, which promotes the transition of TAM M2 (tumorigenic TAM) to a TAM M1 subtype. In an attempt to increase oxygen nanocarrier delivery to colon cancer cells, He and collaborators reported the development of an AMH core–shell gold nanoplateform coated with MnO_2_ and HA for targeted delivery in colorectal tumors and immunogenic phototherapy stimulated by oxygenation in situ. These AMH oxygen-generating nanophotosensitizers were found to trigger apoptosis, CALR exposure, and DC maturation in vitro, as well as release MnO_2_ in the microenvironment of CT26 tumors upon NIR irradiation, thus triggering sufficient oxygen production to relieve tumor hypoxia and inducing a peritumoral immune response in vivo [98]. Shao et al. designed a core–shell heterostructure combining a porphyrinic metal–organic framework (MOF) as the shell and individual lanthanide-doped upconversion nanoparticles, called UCSs. Singlet oxygen generation was observed upon 980 nm light irradiation of UCSs. UCS-based PDT exhibited a significant in vitro cytotoxicity on CT26 mouse colon cancer models, which was further increased in hypoxic growing conditions when the tirapazamine (TPZ) hypoxia-activable prodrug [99] was loaded on the nanoplateform. Application of CT26 grafts to immunocompetent mice models with TPZ/UCSs showed a remarkable antitumor effect upon NIR irradiation and synergized with anti-PD-L1 immunotherapy, with the promotion of a robust abscopal effect that completely inhibited the growth of distant untreated tumors by generating tumor infiltration specific to cytotoxic T lymphocytes [100].

Most of the studies cited above used bilateral subcutaneous tumor models as a metastatic model and drew conclusions about the antimetastatic effect of PDT based on the observation of an abscopal effect. Some other studies used cell lines (e.g., the 4T1 breast cancer cells) that naturally spread to distant sites. However, these models are unlikely to reflect the molecular evolution that can be found in patients between primary tumors and metastatic clones that spread to different organs, which might respond differently to the same therapy [101]. At this stage, this should be taken into account while interpreting these observations. Furthermore, animal models are also unlikely to recapitulate the situation in patients, since the abscopal effect upon treatment with ICIs in patients has been proposed to be a rare event [102].

## 5. Perspectives

Metal-based PSs have a promising potential for application as PDT-based anticancer drugs. More particularly, their ability to induce an inflammation and modulate the tumor immune microenvironment shows that they may be used in synergy with ICI immunotherapies. However, several limitations or obstacles need to be overcome in the future to accelerate their transfer to routine tumor management. Some of these limitations, including water solubility and optimal maximal excitation in the NIR region, can be tackled through the design of innovative chemical complexes by combining metals to new ligands that might enhance the performances of the PSs. Targeted delivery of PS to tumor cells is also an important goal to achieve in order to avoid sun/light sensibility, which remains a major drawback of PDT, limiting a broader application. Targeted PS delivery could, therefore, be achieved by using bioconjugates where metal-derived compounds are complexed to antibodies that target tumor antigens used in the clinic (e.g., the cetuximab anti-epidermal growth factor receptor (EGFR) or the trastuzumab anti-human epidermal growth factor receptor-2 (HER-2) antibodies). In vivo data, which are generally missing, will confirm whether this is a viable option. Lastly, to properly use PDT in combination with immunotherapy for a personalized therapy, a more detailed understanding of the mode of action of metal-based PDT compounds is required. For instance, the molecular mechanisms that underlie the induction of ICD upon metal-based PDT, especially the signaling pathways that are functionally required for the emission of DAMPs, are still ill-defined. More specifically, it is still not clear whether the induction or the ER stress is functionally necessary and/or sufficient to trigger the plasmic membrane relocalization of ER-resident chaperones. In addition, the immune landscape of the tumor microenvironment is both complex and heterogeneous; it is composed of a large repertoire of immunosuppressive (responsible for tolerance) and cytotoxic (responsible for cancer cell elimination) infiltrating immune cells, in their respective abundances, showing complex interactions through the secretion of cytokines and chemokines. It remains to be determined whether the composition of a tumor immune landscape impacts the therapeutic efficiency of metal-based PDT, and to what extent PDT-related ICD influences this tumor immune microenvironment. Thorough research programs that address these issues must be carried out in order to not only gain a better comprehension of these phenomena, but also uncover potential resistance mechanisms and additional synergistic therapeutic approaches. These efforts might eventually help PDT based on metal PSs to cross the gap from bench to bedside.

## Figures and Tables

**Table 1:** Cancer indications for PS-based PDT.

Indication	PDT-PS	Refs.
Obstructing esophageal cancer	Photofrin II	[24]
Non-small-cell lung cancer	Radachlorin^®^	[25]
Recurring head and neck squamous cell carcinoma	Temoporfin/Foscan^®^	[26]
Localized prostate cancer	Padeliporfin/TOOKAD^®^	[27]
Superficial basal-cell carcinoma	Methylaminolevulinate/Metvix^®^	[28]

## Data Availability

Not applicable.
